# Climate change linked to vampire bat expansion and rabies virus spillover

**DOI:** 10.1111/ecog.06714

**Published:** 2023-10-26

**Authors:** Paige Van de Vuurst, Huijie Qiao, Diego Soler-Tovar, Luis E. Escobar

**Affiliations:** 1 Virginia Tech Graduate School, Translational Biology, Medicine, and Health Program, Blacksburg VA, USA; 2 Department of Fish and Wildlife Conservation, Virginia Tech, Blacksburg, VA, USA; 3 Institute of Zoology, Chinese Academy of Sciences, Beijing, China; 4 Facultad de Ciencias Agropecuarias, Universidad de La Salle, Bogotá, Colombia; 5 Global Change Center, Virginia Tech, Blacksburg, VA, USA; 6 Center for Emerging Zoonotic and Arthropod-borne Pathogens, Virginia Tech, Blacksburg, VA, USA

**Keywords:** bats, climate change, emerging infectious disease, rabies, spillover

## Abstract

Bat-borne pathogens are a threat to global health and in recent history have had major impacts on human morbidity and mortality. Examples include diseases such as rabies, Nipah virus encephalitis, and severe acute respiratory syndrome (SARS). Climate change may exacerbate the emergence of bat-borne pathogens by affecting the ecology of bats in tropical ecosystems. Here, we report the impacts of climate change on the distributional ecology of the common vampire bat *Desmodus rotundus* across the last century. Our retrospective analysis revealed a positive relationship between changes in climate and the northern expansion of the distribution of *D. rotundus* in North America. Furthermore, we also found a reduction in the standard deviation of temperatures at *D. rotundus* capture locations during the last century, expressed as more consistent, less-seasonal climate in recent years. These results elucidate an association between *D. rotundus* range expansion and a continental-level rise in rabies virus spill-over transmission from *D. rotundus* to cattle in the last 50 years of the 120-year study period. This correlative study, based on field observations, offers empirical evidence supporting previous statistical and mathematical simulation-based studies reporting a likely increase of bat-borne diseases in response to climate change. We conclude that the *D. rotundus* rabies system exemplifies the consequences of climate change augmentation at the wildlife–livestock–human interface, demonstrating how global change acts upon these complex and interconnected systems to drive increased disease emergence.

## Introduction

Human disturbance of ecosystems can modify wildlife–pathogens dynamics, which can result in the emergence of infectious diseases (Morens et al. 2004). Interactions among humans, livestock, and wildlife populations associated with ecosystem disturbance can facilitate pathogen spillover (i.e. cross-species pathogen transmission) (Plowright et al. 2017). A series of factors have been proposed as facilitators of pathogen spillover from wildlife to humans; specifically land use change (Gibb et al. 2020). Nevertheless, one area of scientific uncertainty and concern is the potential impact of global climate change on wildlife pathogen spillover (Carlson et al. 2022). Climate change can have comprehensive impacts across human, animal, and ecosystem health, potentially acting as a key driver of host–pathogen interaction frequency (Arthur et al. 2017, Cohen et al. 2019, Carlson et al. 2022). Many climate-change studies on spillover risk, however, have been based on future climate change simulations, rather than retrospective empirical data (Carlson et al. 2022). Additionally, little climate-change research has been conducted on directly transmitted diseases of wildlife origin. Infectious agents such as Hendra virus, Middle East respiratory syndrome coronavirus (MERS-CoV), and Nipah virus have emerged to cause pathogenic outbreaks in humans via spillover transmission from bats to intermediate animal hosts (Letko et al. 2020). The mechanisms of spillover transmission, successful establishment in new species, and how posterior spread continues on to cause epidemics are vital components of the broader disease emergence continuum. As such, understanding the extent to which climate change influences bat ecology is of critical importance for the understanding and prediction of emerging bat-borne diseases.

Rabies virus (RABV) belongs to the genus *Lyssavirus* (Rhabdoviridae), which causes rabies disease. Bat-borne RABV has been reported to only occur in the Western Hemisphere (Velasco-Villa et al. 2017b). Rabies is one of the oldest directly transmitted infectious diseases to affect humans in recorded history and the most lethal of all zoonotic diseases, with fatality rates at nearly 100% (Meske et al. 2021). Approximately 50 000 recorded human deaths due to rabies are reported annually despite vaccination efforts, with many of these deaths occurring in at-risk populations (Hampson et al. 2015). Rabies also has precipitous impacts to livestock, domesticated pets, and wildlife (Meske et al. 2021). Bat-borne RABV spillover from wildlife to domesticated species is frequent and widespread in the Americas (Pan American Health Organization 2022). The common vampire bat *Desmodus rotundus*, one of three sanguivorous species in the Phyllostomidae family, is considered to be the main wildlife species responsible for transmitting RABV to other domestic animals and humans in Latin America (Meske et al. 2021).

*Desmodus rotundus*-transmitted RABV is a well understood and documented directly transmitted pathogen of wildlife origin in tropical and sub-tropical regions in Latin America, where RABV is commonly transmitted from *D. rotundus* to cattle (Pan American Health Organization 2022). Recent years have seen an increase in rabies transmission in Latin America, with over 1500 animal cases being reported in 2021 alone (Pan American Health Organization 2022). Temperature limitations on *D. rotundus* occurrence have been recorded in previous literature, owing to the species’ physiology (Lyman and Wimsatt 1966, McNab 1973, Arellano-Sota 1988b, Flores-Crespo and Arellano-Sota 1991). *Desmodus rotundus* is a homeothermic species which has demonstrated a varied response to extreme temperatures in laboratory settings (Lyman and Wimsatt 1966). It is hypothesized that *D. rotundus* individuals have poor thermoregulation at temperatures outside their optimal tolerances (average temperatures between 21 and 28°C) (McNab 1973, Arellano-Sota 1988a, Flores-Crespo and Arellano-Sota 1991). As such, climatic changes could contribute to changes in *D. rotundus* distribution across time.

Debate still exists within the literature regarding the potential response of *D. rotundus* to climate change, including whether or not the species could extend its range into currently temperate regions in the future. Previous assessments concluded that a range expansion of *D. rotundus* into the USA was unlikely, owing to elevated temperature seasonality in temperate areas (Lee et al. 2012). Nevertheless, another study found that *D. rotundus* could very likely expand its range northward through routes in southern Texas (Hayes and Piaggio 2018). *Desmodus rotundus* range expansion could contribute to the invasion of the species into the southern US, thereby bringing with it an increased risk of rabies emergence in new regions. The disagreements between previous modeling efforts have shown that further study is still warranted within this disease system. We assessed *D. rotundus* annual distributions following climatic variation from the last century using data-driven ecological niche modeling. We also assessed the current scope and temporal pattern of rabies outbreaks in cattle across Latin America to identify if *D. rotundus* distribution is related to spillover frequency in this system.

## Material and methods

To assess the impact of past climatic variation on the distribution of *D. rotundus,* we utilized ecological niche modeling methods to develop a multidimensional timeseries analysis of the species’ geographic range across the last century. Ecological niche models were done using a presence-background modeling approach, which does not require absence data and therefore more logically abides by the available occurrence data for this species. The ecological niche modeling methods used contrasts the environmental conditions associated with species’ presences points (i.e. occurrence points) with randomly selected background points from the available environmental space where the species could potentially occur (i.e. study area extent).

### Climate data

To summarize retrospective climatic variability we used six representative climate variables from the climatic research unit gridded time series (CRU TS) ver. 4.04 database at 0.5° latitude by 0.5° longitude resolution from 1901 to 2019 (Harris et al. 2020). To reduce dimensionality in the final modeling effort and to summarize annual climatic variability we collated the available monthly bioclimatic variables from CRU TS into annual level rasters. Representative climate variables included: average temperature, temperature standard deviation, average diurnal temperature range, average cloud cover, cloud cover standard deviation, and potential evapotranspiration standard deviation. These variables were converted to ASCII format and were used at the annual level for the occurrence data filtering, model calibration, and model projection process.

### *Desmodus rotundus* occurrence data and filtering

Occurrence records from 1901 to 2019 were sourced from a previously curated *D. rotundus* occurrence dataset, which contains comprehensive data from a variety of sources (Van de Vuurst et al. 2021). These include publicly available resources and databases in the scientific literature, natural history museums across North, Central and South America, and official records from ministries of agriculture and health (Van de Vuurst et al. 2021). To address possible sample selection bias and spatial autocorrelation, we resampled *D. rotundus* occurrences to one per pixel of the environmental layers. We then utilized the environmental background data to identify and remove outliers in environmental space (Peterson et al. 2011). The values of each climatic variable were first extracted from the location and year of each occurrence (between 1901 and 2019) to create a cloud of data points representing the species distribution in environmental space (Peterson et al. 2011). For occurrence records which had age or life stage metadata, we extracted the variable values from multiple years based upon the age of the individual to account for persistent occupancy at that location. For juvenile individuals, only the singular year of occurrence was extracted from the corresponding annual raster. For individuals that were classified as adults, we extracted the year the occurrence was recorded and the four years prior (five years total). Age delineations were based on previous *D. rotundus* capture data (Lord et al. 1976), which indicates that most captured adult *D. rotundus* individuals are less than six years of age. We then developed a principal component analysis of the six climatic variables to obtain principal component axes which summarized the variance of the data. Principal components one, two, and three (summarizing 82.9% of the data variance) were then used as axes (i.e. X, Y and Z axes) to plot the species occurrence records in three environmental dimensions. We then calculated the Mahalanobis distance between occurrence points in the principal component environmental space. We fit a minimum volume ellipsoid to the distribution of points within the principal component space (PCs one, two and three) using on point per quantile of the spatially filtered points (one per pixel of the study extent) with a precision factor of one (i.e. interval of 0.99). Finally, we used a χ^2^ test to identify environmental outliers (i.e. those points which fell outside of the ellipsoid) from the cloud of extracted values, which were removed from the analysis (Supporting information). The remaining filtered occurrences were randomly split into 50% training and 50% testing subsets from the thinned dataset for each model calibration and evaluation replicate.

### Model calibration and evaluation

For this modeling effort, we used a presence-background ecological niche modeling algorithm based on maximum entropy (Maxent ver. 3.4.4) (Warren and Seifert 2011, Phillips et al. 2019) within the ‘kuenm’ package in R statistical software (www.r-project.org, Cobos et al. 2019). ‘kuenm’ is an R package designed to make the process of model calibration and final model creation more reproducible and robust (Cobos et al. 2019). Using ‘kuenm’, we created suites of candidate models with various parameterizations including diverse feature and regularization combinations. Model overfitting is minimized in Maxent by the use of functions derived from the environmental variables (i.e. feature classes) and regularization parameters which impose penalties on the model for over-complexity (Phillips and Dudík 2008, Morales et al. 2017). We tested a suite of regularization parameters (0.1–1.0 by increasing values of 0.1, 2–6 by values of 1, 8, and 10) and all twenty-nine possible combinations of five feature classes (linear = l, quadratic = q, product = p, threshold = t, and hinge = h) to ensure all possible models were considered. After redundant model combinations were removed, this resulted in 1054 candidate models for each replication. The candidate model formation, evaluation, and best model selection process was repeated 100 times (for a total of 105 400 models) to increase confidence in the resulting best model features validity via statistical replication. The best model from each replicate was reported and its regularization multipliers and feature classes were recorded. The best candidate model regularization multiplier and feature classes that appeared most often from the 100 replicates were then selected for the final model projection process.

Candidate models were evaluated using the *kuenm_ceval* function. Candidate model performance evaluation was based on significance (partial ROC, with 100 iterations and 80 percent of data for K-fold validation), omission rates (*E* = 5%), and model complexity and fit to the calibration data (i.e. Akaike information criterion, AIC) (Hobbs and Hilborn 2006). Both partial ROC and omission rate were used as preliminary measures to identify models which were significantly better than random. AICc was used as the delineating value of best model selection, with all models of delta AICc > 2 being excluded, as AICc is a more meaningful measure of model performance (Lobo et al. 2008, Peterson et al. 2008, Warren and Seifert 2011). This model selection process also allowed us to identify the best parameterization (i.e. combination of feature classes and regularization multipliers) for our model projections. The final model was then used to project the *D. rotundus* range across the entire temporal extent of the study (1901–2019).

### Distributional shift and rabies outbreak assessment

**Figure 1.**
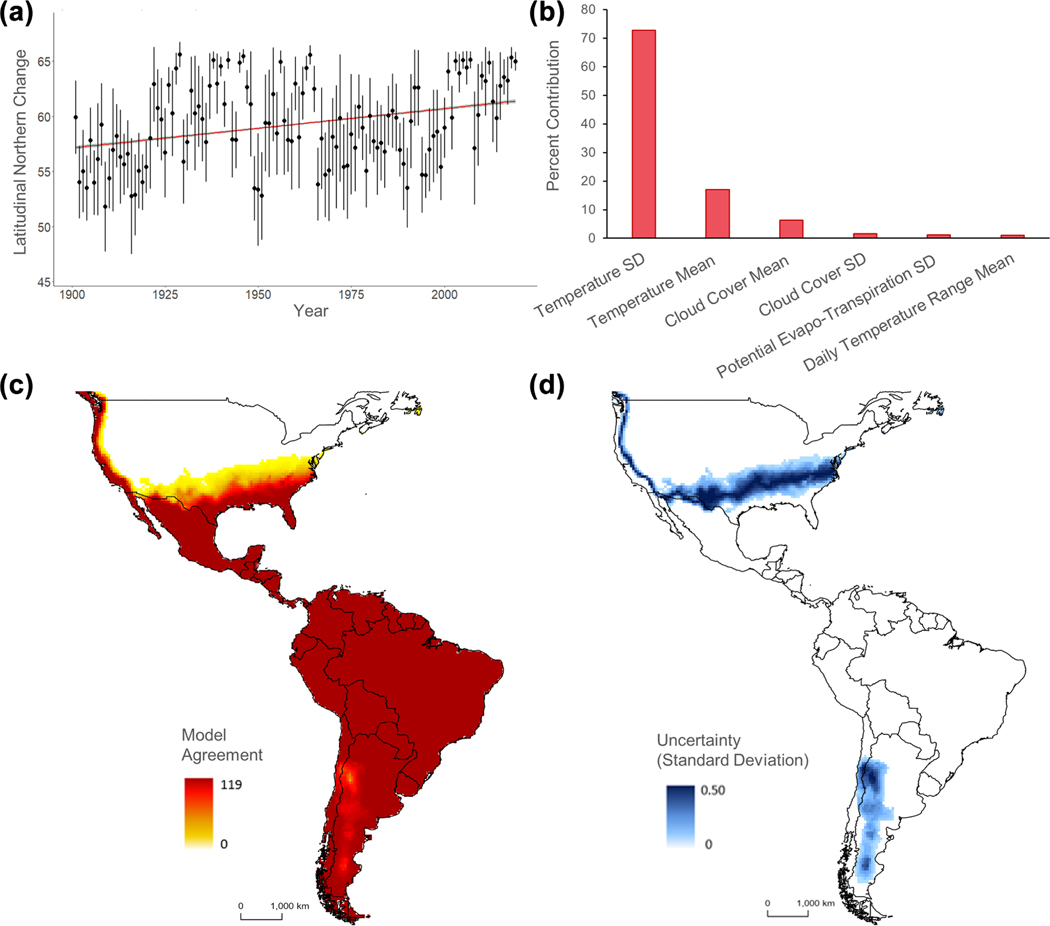
Range shift of *Desmodus rotundus* due to climate change. (a) Predicted northward trend (based on ecological niche models) of *D. rotundus* range (slope = 0.04, *R*^2^ = 0.06, p < 0.001). (b) Estimates of relative percent contributions of the environmental variables on the distribution of *D. rotundus*. (c) Model ensemble of *D. rotundus* distributions from 1901 to 2019. (Darker colors indicate higher agreement in models regarding *D. rotundus* distributions.) (d) Uncertainty map revealing areas with higher uncertainty (darker colors of blue) with regard to the potential areas of *D. rotundus* expansion.

The minimum training presence value from the final model was used as a threshold to reclassify the projected range maps into annual binary maps. Based on the assumption that the least suitable environment at which the species is known to occur is the minimum suitability value recorded in the calibration dataset, this threshold can be used to delineate areas of possible species range from areas where it is unlikely for the species to occur. The resulting binary maps were used to assess how the projected suitable range for *D. rotundus* has changed across time. We used the *cellStats* function of the ‘raster’ package (Etten et al. 2021) in R to quantify the total range area from each binary map. We then isolated the 100 highest (most northern) and lowest (most southern) latitudes predicted by the projected range models, which allowed us to assess whether or not the projected suitable range for *D. rotundus* was moving northward or southward across time based upon our model (Fig. 1). We also assessed modeled range expansion using a generalized additive model (GAM) to determine if nonlinear trends of distributional change across time were present (Supporting information). Additionally, we identified the 100 highest projected suitable elevations from each annual binary map and averaged these values across time to determine the extent to which *D. rotundus*’ suitable range varied in elevation. Elevation data were collected from the WorldClim bioclimatic variable database at five arc minute resolution (approx. 10 km) in raw agreement with the climate data (Fick and Hijmans 2017). We used linear models to assess the relationships between time as measured by year and the three resulting suitability characteristics (i.e. area, elevation, and latitude of observed and predicted distribution).

To identify range shift rates of observed *D. rotundus* occurrence across time (i.e. from recorded occurrence points and not from modeled range projections) we identified the 20 most northern occurrence points of the species for the occurrence database (Van de Vuurst et al. 2021) for each year using the ‘dplyr’ package in R (Wickham et al. 2020). This allowed us to ascertain the average most northern and most southern extent of recorded species occurrence for each year. We then calculated the average change in latitude by decimal degrees per year using the ‘dplyr’ package (Wickham et al. 2020), and converted this result to kilometers using the assumption that one decimal degree is approximately 111 km. We used this metric to identify how long it may take *D. rotundus* to appear in the USA by dividing the distance between the US-Mexico border and the most northern recorded occurrence of the species by the range shift rate. We also subset the original occurrence dataset to isolate *D. rotundus* occurrences from Mexico. We identified the temperature standard deviation of each occurrence from Mexico corresponding to the year of the capture location from the CRU database, and utilized a linear model to identify how temperature standard deviation has changed across the study period.

Data on the presence of bovine rabies cases transmitted by *D. rotundus* were collected from the Regional Information System for the Epidemiological Surveillance of Rabies (SIRVERA) (PANAFTOSA 2021). SIRVERA is a database for the prevention of rabies in the Americas, where countries report the presence of rabies on a monthly basis (Belotto et al. 2005, Seneschall and Luna-Farro 2013, Vigilato et al. 2013, Del Rio Vilas et al. 2017, Velasco-Villa et al. 2017a, Freire de Carvalho et al. 2018, Benavides et al. 2019, Rysava et al. 2020). SIRVERA is made up of more than 54 000 records, most of which are bovine in origin. Of 29 countries in the Americas in the system, the largest number of reports originate from Brazil, Mexico, and Peru. The data were filtered according to the following criteria: 1) types of cases: animal cases, 2) date of notification: January 1970–December 2020, 3) variant: genetic variant three of the rabies virus (specific to *D. rotundus*), 4) species: bovine (species of domestic animal most affected), and 5) aggressor species: sanguivorous bat (refers to the common vampire bat or *D. rotundus*).

Effect of *D. rotundus* range change on RABV spillover transmission was assessed based on data availability for RABV records between the 1970s (1970–1980) and 2010s (2010–2020). The ‘dplyr’, ‘sp’, ‘rgdal’, and ‘ggplot2’ packages of the software R (ver. 4.1.0) and RStudio (ver. 2022.02.3) were used (Wickham et al. 2020). We also used the SIRVERA database to isolate rabies cases in domesticated livestock and companion animals from 1970 to 2019 and used the aggregate function in R software to identify the number of rabies outbreaks per year from all countries in Latin America. We then assessed the relationship between the number of rabies outbreaks per year and the projected northern range expansion per year from our ecological niche model. We assessed this relationship using a linear model and displayed the resultant model using ‘ggplot2’.

## Results

Of the 100 replications of the model calibration and evaluation process, the most selected feature class combination was linear, product, quadratic, and hinge, and the most selected regularization parameter was 0.2. These features and parameter were used for the final model and projection efforts. Of the six environmental variables used, standard deviation of temperature contributed the most to the final Maxent model (72.7%), followed by average temperature (17%) and cloud cover mean (6.3%). The remaining variables (cloud cover standard deviation, potential evapotranspiration standard deviation, and average daily temperature range) combined contributed less than 10% to the final model. We found no significant change in the total area of *D. rotundus* distribution during the 120-year period assessed (slope = 0.39, *R*^2^ = 0.03 × 10^−2^, p = 0.31). We also found no significant trend of elevational shift for the species’ range (slope = 0.03, *R*^2^ = −5.74 × 10^−5^, p = 0.57). Nevertheless, we did find that *D. rotundus’* geographic range has significantly shifted its distribution northward (slope = 0.04, *R*^2^ = 0.06, p < 0.001) (Fig. 1), which represents a natural invasion into northern Mexico at an average rate of 9.76 km per year (standard error = 1.03 km). At this speed, *D. rotundus* could extend its range into the continental USA in the next 27 years. These results were echoed in our GAM of range expansion northward (*R*^*2*^ = 0.21, p < 0.01) (Supporting information). We did not identify any significant trend of range expansion to the south within *D. rotundus’* range (slope = −0.04, *R*^2^ = 0.03, p = 0.12). We found areas of model uncertainty along high-lands in the Andes Mountains and in temperate portions of the USA (Fig. 1).

**Figure 2.**
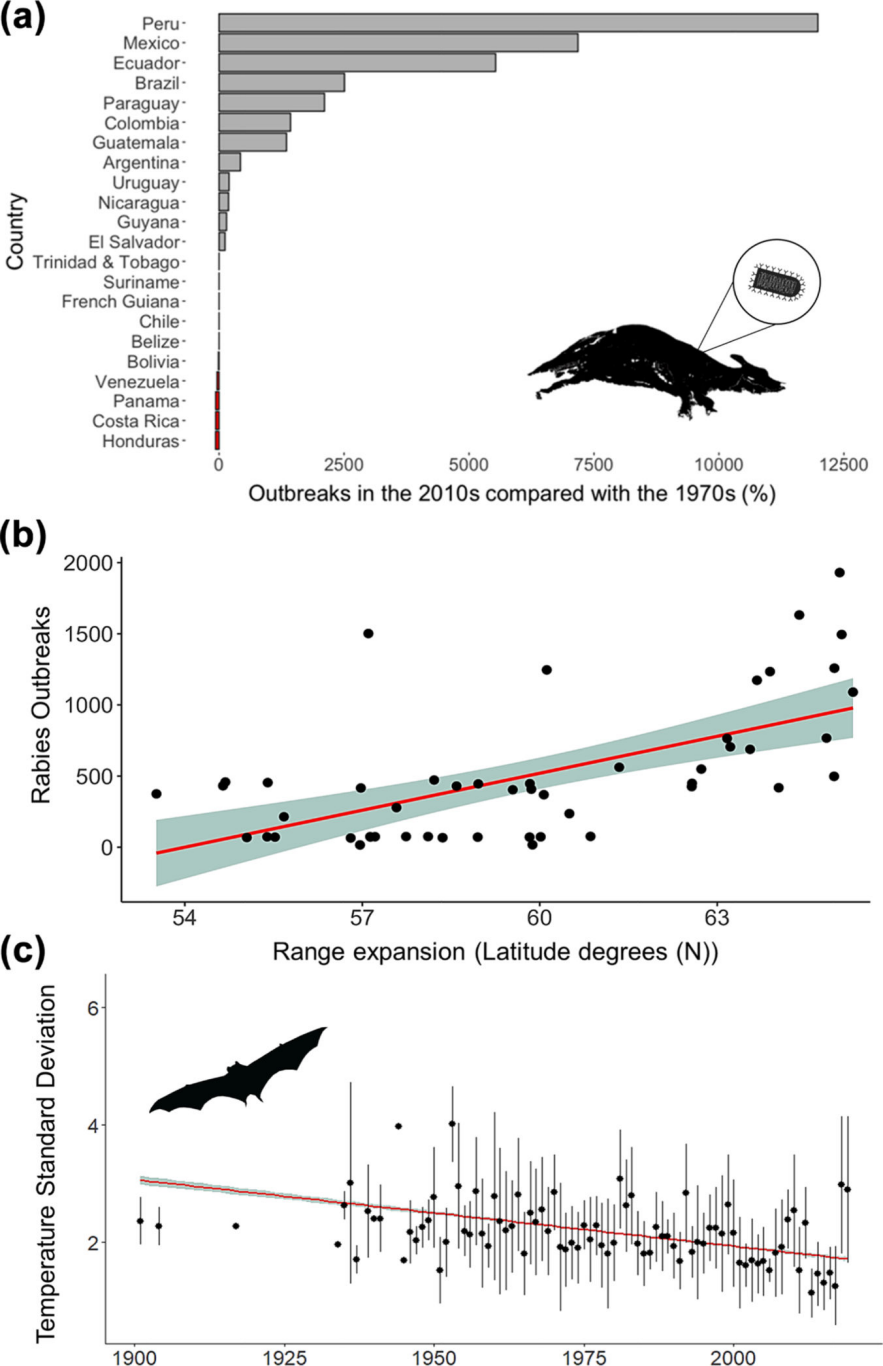
Bat-borne rabies outbreak increase in Latin America. (a) Percentage change in rabies outbreaks in cattle for Latin American countries between the 1970s and 2010s. (b) Regression relationship between northern range expansion of *Desmodus rotundus* and outbreaks of rabies in Latin America (1970–2019). (Slope = 86.61, *R*^*2*^ = 0.36, p < 0.001.) (c) Reduction in temperature seasonality in *D. rotundus* capture locations across time. (Slope = −0.01, *R*^2^ = 0.07, p < 0.001.) Black points and lines: distribution of temperature standard deviations of all *D. rotundus* occurrence locations from each corresponding year.

The climatic variable that most influenced the range shift in *D. rotundus* distribution was historical temperature seasonality (i.e. standard deviation), a variable closely linked with changes in climate. At the northernmost extent of *D. rotundus’* range (Mexico), we found that temperature seasonality of *D. rotundus* capture sites has significantly decreased across time (slope = −0.01, *R*^2^ = 0.07, p < 0.001; Fig. 2). We also detected a linear positive increase in the number of rabies outbreaks in the last 40 years (slope = 23.25, *R*^*2*^ = 0.49, p < 0.001), and *D. rotundus* range expansion (slope = 0.15, *R*^2^ = 0.38, p < 0.001) (Supporting information). The respective increases in rabies outbreaks and *D. rotundus* range expansion were positively associated (slope = 86.61, *R*^2^ = 0.36, p < 0.001) (Fig. 2). By the 2010s, the number of rabies outbreaks in cattle increased in most Latin American countries from 1000 to 12500% compared to a 1970s baseline. The largest increases in the number of rabies outbreaks across time occurred in Peru, Mexico, Ecuador and Brazil (Fig. 2).

## Discussion

Results revealed an increase in bat-borne RABV spillover transmission in the last 40 years. Similarly, the century-long *D. rotundus* occurrence data assessment shows a relationship between climate change and the distribution of the RABV reservoir across time. Based on this evidence, ongoing climate change is linked to distributional shifts of *D. rotundus* in tandem with continental-level changes in the risk of RABV spillover from wildlife to domestic animals. As such, this analysis provides a prime example of retrospective climate change-driven range shifts of a bat reservoir, and the pathogen it transmits, which until now has been more commonly associated with future-climate simulation-based models. The range shift signals detected echo current expert opinion anticipating *D. rotundus* invasion into the southern USA within the next 5–20 years (Davis and Chipman 2020). We did not detect a significant southern extension of the range of *D. rotundus* into new areas of Chile and Argentina, potentially due to the model uncertainty identified in this region (Fig. 1). An imperceptible range expansion or contraction in the most southern areas of the species’ distribution could be linked to less marked climatic variation in the regions, as compared to the northern part of the continent. Additionally, our lack of signal in the southern part of *D. rotundus’* range could be due to a low density of occupancy in the south, which could reduce their probability of detection, limiting the tractability of changes in the species’ distribution across the Southern Hemisphere. Alternatively, there may not be continuous and abundant cattle populations across the southern region of the species’ range (i.e. resources) to facilitate climate-driven range expansion. Nevertheless, our findings clarify previous inconclusive future-climate forecasts of *D. rotundus* distributional changes in response to climate change (Lee et al. 2012, Zarza et al. 2017, Hayes and Piaggio 2018).

Previous explorations on *D. rotundus* distribution conducted with more modest datasets and modeling effort (Lee et al. 2012, Zarza et al. 2017) also found that minimum temperatures and seasonality (i.e. differences between coldest and warmest seasons) are among the major drivers of *D. rotundus* distribution. Previous research has suggested that *D. rotundus* may not survive well in environments with temperatures below 15°C (Arellano-Sota 1988b). For example, it is assumed that *D. rotundus* individuals cannot consume enough blood nightly to effectively thermoregulate below 15°C (Lyman and Wimsatt 1966, McNab 1973). It is worth noting, however, that the cited survival assessment was based on a smaller sample size in a laboratory, and a large degree of variation in the tolerance of cold has been observed among *D. rotundus* individuals in other assessments (Wimsatt 1962, Lyman and Wimsatt 1966). *Desmodus rotundus* has also been recorded in cooler environments in more recent studies (Hilaire et al. 2019). Temperature-related limitations could have also contributed to the uncertainty observed in high elevation landscapes such as the Andes (Fig. 1). A more in-depth assessment of the true minimum temperature limitation for *D. rotundus* occupancy in the wild could elucidate the uncertainties identified in this study, and could present an avenue for future research efforts. Furthermore, climate change not only leads to increases in temperature, but also alterations in annual seasonality (Steltzer and Post 2009, Wang et al. 2021). As such, the reduction in temperature monthly variation at sites with *D. rotundus* records in the most northern extents of their range (Fig. 2) supports the postulation that climate change may be driving range extension by making temperature more stable across the year.

One limitation to our analysis is the use of aggregated country level RABV data, which innately limits the ecological applicability of our assessment to broader spatial and ecological scales. Future studies could utilize finer scale data at the locality scale to identify local factors that contribute to *D. rotundus* range expansion and spillover transmission. An important caveat of our modeling effort is the inclusion of only abiotic climatic variables. While abiotic variables were sufficient to answer our biogeographic question as to the role of climate in driving the spatiotemporal distribution of *D. rotundus*, it is possible that biotic variables, such as local anthropogenic disturbance, may have contributed to the range expansion of *D. rotundus* (Soberón and Nakamura 2009, Streicker et al. 2012). For example, an increase in cattle density, associated landscape changes, or biodiversity loss may drive greater contact between livestock and *D. rotundus* individuals in Latin America (Streicker et al. 2012, Domenici et al. 2023). Future research could assess the effects of biotic variables, such as prey availability and composition (Brown and Escobar 2023), on the ecology of *D. rotundus* at finer scales. Additionally, we assumed a linear relationship between time and distributional trends of *D. rotundus* to mitigate overfit of more complex models. Nevertheless, patterns identified utilizing non-linear models were also significant (Supporting information). The bulk of climate change and disease research is influenced by future climate simulation modeling studies and biased towards vector-borne diseases (Van de Vuurst and Escobar 2023). As such, this study is among the first data-driven evidences of climate change effects on the risk of spillover transmission of a zoonotic pathogen of bat origin. Species range shifts may modify species assemblages (Bertrand et al. 2011, Pecl et al. 2017), which in turn could increase the risk of sharing pathogens and parasites between native and invasive species (Carlson et al. 2022). Although species’ range shifts are considered a major response of biodiversity to climate change (Sunday et al. 2012, Williams and Blois 2018), we are in the infancy of understanding how climate change-induced range shift will affect the burden of bat-borne infectious disease. Early empirical evidence of climate change, species range shifts, and zoonotic diseases includes the increase in transmission of vector-borne diseases in historically cold regions, which are now becoming suitable for vectors (Siraj et al. 2014, Ryan et al. 2019). For instance, malaria has been recently linked to temperature change in the highlands of Colombia and Ethiopia (Siraj et al. 2014). Nevertheless, the effects of climate change on directly transmitted (not vectorborne) diseases are less studied. Future research is warranted to assess the retrospective impact of climate change on other wildlife pathogens of pandemic potential.

*Desmodus rotundus* RABV at the US-Mexico border threatens both public health and the livestock industry in the southern USA (Bodenchuk and Bergman 2020, Davis and Chipman 2020). For instance, in Latin America, most rabies cases in humans and domestic animals originate from bites by *D. rotundus*, which feeds on the blood of vertebrate animals (Velasco-Villa et al. 2017a,b). RABV transmission from *D. rotundus* is endemic in Latin America, where it generates considerable and widespread death of livestock and humans (Benavides et al. 2020). This study reveals that *D. rotundus* is expanding its distribution to higher latitudes, which shows the potential of the species to become invasive in novel areas and new countries. In 2016, the US Department of Agriculture (USDA) documented the first occurrence of *D. rotundus* near the US-Mexico border (Keirn 2016, Piaggio et al. 2017, Hayes and Piaggio 2018). Furthermore, genetic assessments have demonstrated that *D. rotundus* from Mexico is expanding its range northward rapidly (Piaggio et al. 2017), but the likely drivers of such geographic expansion were unknown, until now. Range shift of *D. rotundus* is considered the cause of the increased geographic distribution and incidence of *D. rotundus* RABV transmission in most of Latin America (Benavides et al. 2016, 2020, Piaggio et al. 2017, Botto Nuñez et al. 2019). In 2020, USDA released notes of concern regarding the risks of *D. rotundus* RABV spread to the USA (Bodenchuk and Bergman 2020, Davis and Chipman 2020).

The ongoing spread of *D. rotundus* RABV to new countries, including the USA, is problematic for livestock production and public health. If not treated promptly, RABV infections in humans are 100% fatal (Rupprecht et al. 2002). In the Americas, rabies is a zoonosis with a complex epidemiology, involving the virus circulating in wildlife, including bats. In the USA, more than 90% of rabies cases originate in wildlife (Blanton et al. 2010). Bats that feed on insects have the highest RABV incidence in wildlife in the USA (33% of all animal cases in 2018), followed by raccoons (30.3%), skunks (20.3%), and foxes (7.2%) (Ma et al. 2020). RABV is endemic in wildlife in the USA, where every year at least 55 000 humans are exposed to the virus from wildlife, mainly bats (70%) (Pieracci et al. 2019). Exposed human individuals typically receive immediate post-exposure vaccination and in some cases immunoglobulin treatment, which is expensive (often thousands of US dollars per treatment) but which has significantly reduced human mortality (Rupprecht et al. 2002, Pieracci et al. 2019). Nevertheless, bat-borne rabies has been on the rise in recent years (Ma et al. 2020, Gross 2022, Kunkel et al. 2022). Expenditures for RABV diagnostics, prevention, and control in the USA is between $245 and $510 million annually (CDC 2019) and is expected to increase if wildlife RABV from Latin America spreads to the USA (Anderson and Shwiff 2014). Indeed, arrival of *D. rotundus* to the southern USA (e.g. Texas and Arizona) would result in an estimated annual economic cost of $7–9 million just in terms of the death of livestock from rabies in south Texas (Anderson and Shwiff 2014).

Our results highlight the potential impacts of climate change on the ecology of domestic-wildlife diseases in tropical ecosystems. Tropical ecosystems have been poorly studied in terms of global change effects on biodiversity (Barlow et al. 2018). By documenting how *D. rotundus* is expanding its range annually, this article can help guide strategies to prevent *D. rotundus* RABV spillover to humans and domestic animals and help prevent the potential spread of *D. rotundus* rabies from Latin America into the USA (Piaggio et al. 2017). This disease biogeography study integrated epidemiological, climatological, and ecological data to enrich the understanding of wildlife RABV spillover across the Americas. Climate change acts as a key driver of the spatial and temporal variation of host–pathogen interaction in Latin America, which could continue under future climate change scenarios. Continued RABV transmission into novel geographic areas could impact human health and economic prospects if the speed and direction of the range expansion are not considered to prevent rabies transmission in currently unaffected areas. In conclusion, the *D. rotundus* RABV system exemplifies the consequences of climate change in the augmentation of disease-spillover risk at the wildlife–livestock–human interface.

## Supplementary Material

Supplementary Material

## Data Availability

Data are available from the Figshare Digital Repository: https://doi.org/10.6084/m9.figshare.24142389 (Van de Vuurst et al. 2023).
