# Supplementary material for: Climate change linked to vampire bat expansion and rabies virus spillover
Source: Ecography. Author manuscript; Available in PMC 2024 Dec 20. (PMC11661686; doi:10.1111/ecog.06714)
Supplement: Supplementary Material [file NIHMS1993048-supplement-Supplementary_Material.docx]

**Supplementary Information**

**
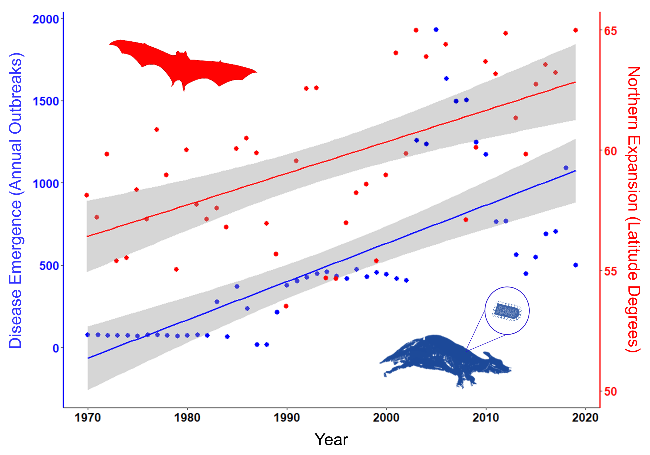
**

**Supplementary Figure 1:** Regression relationship between disease emergence of rabies in Latin America and projected northern expansion of *D. rotundus*. Blue: Linear model of rabies outbreaks across time from 1970-2019 (slope=0.49, *R^2^*=0.49, *p*<0.001). Red: Linear model of maximum projected latitude of *D. rotundus* range across time from 1970-2019 (slope=0.38, *R^2^*=0.38, *p*<0.001).


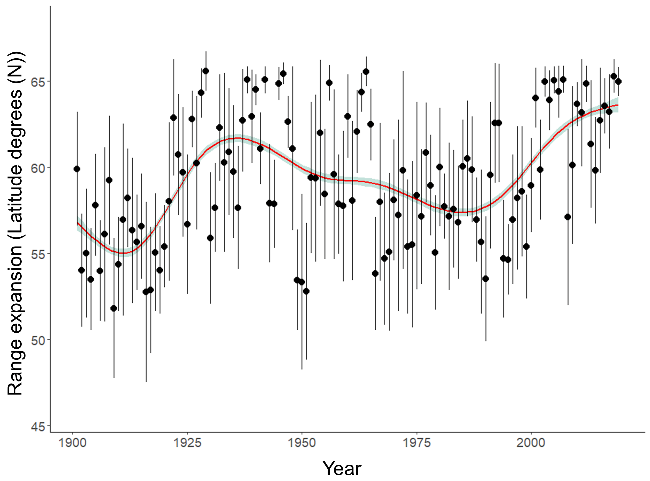


**Supplementary Figure 2:** Relationship between projected northern range expansion (latitude) of *Desmodus rotundus* and time. Dots: median estimated distribution of estimated most northern latitude of *D. rotundus* from each corresponding year. Vertical lines: standard deviation of estimated most northern latitude of *D. rotundus*. Red line: Generalized additive model revealing significant non-linear association between range expansion and year from 1901-2019 (*R^2^=*0.21*, p<0.01*).


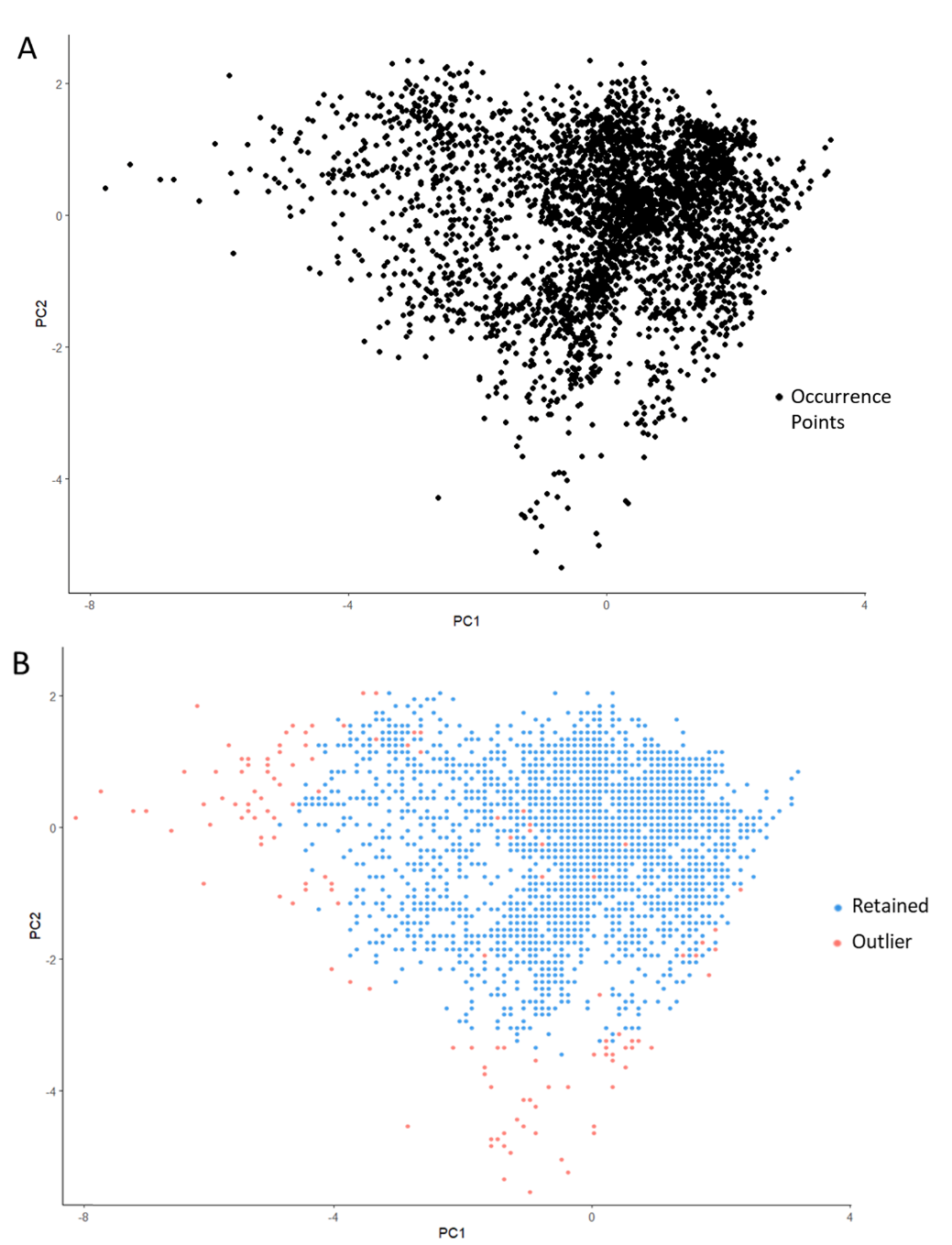


**Supplementary Figure 3:** Principal components one (PC1) and two (PC2) of the environmental variables are used to display original unfiltered *Desmodus rotundus* occurrence points (**A**) and filtered occurrence points (**B**) in environmental space. Occurrence points identified as environmental outliers (red) were excluded from training and testing data subsets while non-outliers (blue) were retained.
